# Systematic identification and characterization of high efficiency Cas9 guide RNAs for therapeutic targeting of *ADAR*

**DOI:** 10.1371/journal.pone.0317745

**Published:** 2025-02-24

**Authors:** Benjamin G. Gowen, Kory Melton, Weng In Leong, Prachi Khekare, Shannon McCawley, Jean Chan, Pierre Boivin, Vihasi Jani, Aaron J. Cantor, Akshay Tambe, Mary Haak-Frendscho, Mary J. Janatpour, Spencer C. Wei

**Affiliations:** Spotlight Therapeutics, Hayward, California, United States of America; University of Surrey, UNITED KINGDOM OF GREAT BRITAIN AND NORTHERN IRELAND

## Abstract

Therapeutic targeting of the adenosine deaminase ADAR has great potential in cancer and other indications; however, it remains unclear what approach can enable effective and selective therapeutic inhibition. Herein, we conduct multi-staged guide RNA screening and identify high efficiency Cas9 guide RNAs to enable a CRISPR/Cas-based approach for *ADAR* knockout. Through characterization in human primary immune cell systems we observe similar activity with two-part guide RNA and single guide RNA, dose responsive activity, similar guide activity rank order across different cell types, and favorable computational off-target profiles of candidate guide RNAs. We determine that knockout of *ADAR* using these guide RNAs induces pharmacodynamic responses primarily consisting of immunological responses such as a type I interferon response, consistent with the known function of ADAR as a key regulator of dsRNA sensing. We observe similar biological effects with targeting only the p150 isoform or both p110 and p150 isoforms of *ADAR*, indicating that at least in the contexts evaluated, loss of p150 ADAR mediates the primary response. These findings provide a resource of well-characterized, high efficiency *ADAR*-targeting Cas9 guide RNAs suitable for genomic medicines utilizing different delivery modalities and addressing different therapeutic areas.

## Introduction

*ADAR* is a gene of great interest to the therapeutic development community with tremendous potential utility both as an RNA editing platform as well as a core node of the regulatory network governing self RNA recognition. ADAR is an adenosine deaminase that mediates A-to-I RNA editing that prevents constitutive and ubiquitous recognition of self RNA. Here we identify and characterize high-efficiency *S. pyogenes* Cas9 guide RNAs (SpCas9 gRNAs) targeting *ADAR* to enable therapeutic gene targeting of this key regulator of RNA biology.

Disruption of the homeostatic function of ADAR leads to engagement of the MDA5-MAVS dsRNA sensing pathways and induction of a type I interferon response [1–3]. This biological response may be harnessed for multiple therapeutic applications. In immuno-oncology, for example, induction of an innate immune response within the local tumor microenvironment (TME) can facilitate the generation of a robust anti-tumor immune response. Evaluation in preclinical models supports this model in which *ADAR* targeting in tumor cells can sensitize response to immune checkpoint blockade treatment [4]. Based on the cell type-independent biological role of ADAR to prevent self RNA recognition, loss of function ADAR is likely to have a similar effect in most cell types - albeit with differential magnitudes of response.

Despite the therapeutic potential of perturbation of ADAR biology there are currently no approved therapies targeting this RNA editing adenosine deaminase. This is, in part, due to multiple properties of ADAR that make it a difficult-to-drug target. Factors contributing to the challenge in modulating specific ADAR activity include the high degree of similarity with paralogs and the shared coding sequence of two isoforms. Conceptually, ADAR (ADAR1) may be inhibited through a variety of methods including chemical inhibition and gene knockout; however, selective inhibition of ADAR (but not its paralogs ADAR2 and ADAR3) has been difficult [5–7]. Two main isoforms of ADAR, p110 and p150, have distinct biological functions. P150 mediates MDA-MAVS activation via IRF3 and NF-kB activation, whereas p110 ADAR mediates MAVS-independent effects including metabolism and transport activities [1–4,8]. In addition to the existence of multiple paralogs of ADAR, the catalytic domain is shared between ADAR p110 and p150, with p110 entirely encoded by p150. Because of these characteristics it is very difficult to inhibit the activity of ADAR and in particular, p150 specifically.

CRISPR-based genome editing technologies have advanced both as powerful research tools as well as for therapeutic approaches since the initial description of CRISPR/Cas9 based genome editing [9–11]. Cas9 is an RNA guided endonuclease that can be utilized for an array of genomic manipulation. The targeting of Cas9 is programmed together by the gRNA protospacer and nuclease-dependent protospacer adjacent motif (PAM). The first CRISPR-based therapy, Casgevy, received FDA approval in 2023 for the treatment of sickle cell disease. CRISPR-mediated gene knockout presents an opportunity to selectively target *ADAR*, and if desired, specifically knockout p150 ADAR while sparing p110 ADAR (note that p110 cannot be specifically targeted with this method given that p110 is entirely encoded by p150). Here we identify high-efficiency CRISPR/Cas9 single guide RNAs (sgRNAs) to support therapeutic knockout targeting of *ADAR*. The methodology we employ to design, screen, and characterize *ADAR*-targeting gRNAs identifies gRNAs for potential therapeutic products compatible with a range of delivery modalities and therapeutic indications, furthers our collective understanding of ADAR biology, and provides an example of an efficient process for therapeutic gRNA identification.

## Results

### In silico identification of *ADAR*-targeting Cas9 guide RNAs

**Fig 1:**
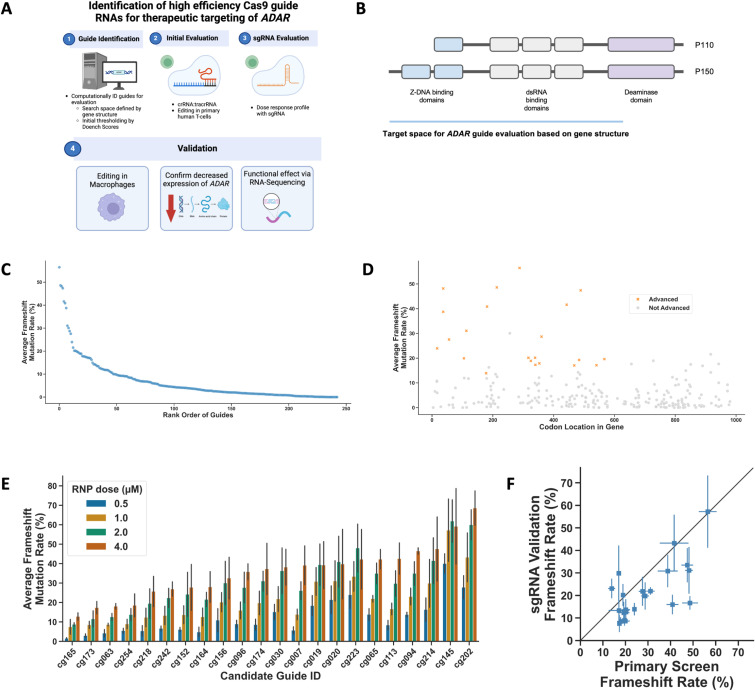
Screening for highly efficient *ADAR*-targeting gRNAs in human T cells. A) Diagram of the workflow used for designing, screening, and validating *ADAR* gRNAs. B) Schematic diagram of the *ADAR* gene, corresponding protein domains, and the target space used for gRNA design. C-D) Results of the *ADAR* gRNA primary screen in human T cells, with gRNAs arranged in rank-order by average frameshift (C) or by location in the gene body (D). Points represent the average of 3 biological replicates. Codon location corresponds to the cut site relative to the coding sequence of the p150 isoform. E) Editing efficiency observed with *ADAR* gRNA secondary gRNA screen in primary human T cells. Guide RNAs are sorted by rank order of the 4 µM dose. Data points represent the average of 3 biological replicates ±  S.D. F) Comparison of frameshift mutation rates observed in the primary vs. secondary screens for the 22 gRNAs chosen for follow-up. Data are plotted as mean ±  S.D.

To identify gRNAs for therapeutic targeting of *ADAR* we defined key criteria to inform selection for empirical evaluation and developed a multi-stage approach for gRNA identification, evaluation, and characterization (Fig 1A, See methods). We defined the genomic search space based on the gene structure and functional characterization of domains. Because we aimed to mediate ADAR loss of function and the deaminase domain is the most C-terminal domain, we identified gRNAs from the translational start site of p150 through the most C-terminal residue reported as required for ADAR deaminase activity (C966) (Fig 1B). We applied a minimum threshold for computational off-target score for gRNAs to proceed to empirical evaluation, given stringent requirements for a therapeutic gRNA (see Methods). Notably, because we sought to identify gRNAs for use in RNP format or other compatible Cas9 delivery modalities (e.g., LNP) we did not impose any restriction based on the ability of the gRNA to be transcribed in mammalian cells. Based on these criteria we selected 243 ADAR-targeting gRNAs for empirical evaluation (S1 Table). To facilitate preclinical evaluation in support of therapeutic development, we assessed the species reactivity of human *ADAR*-targeting gRNAs with mouse, rhesus macaque, and cynomolgus monkey reference genomes (see Methods). 92% (224 of 243) and 4.5% (11 of 243) were reactive as perfect matches with rhesus macaque/cynomolgus monkey and mouse genomes, respectively.

### Primary screen in primary human T cells

Many cell types could be appropriate for evaluating *ADAR*-targeting gRNAs, due to ADAR’s broad expression profile and largely cell-type independent role. We conducted our first level of gRNA screening in primary human T cells, which can be robustly expanded and edited, can be found in the tumor microenvironment, and are relevant to the biology of TME reprogramming [12]. To establish screening conditions suitable for identifying very high-performing sgRNAs, we nucleofected T cells with tool sgRNAs with a range of RNP concentrations, then measured editing at each target locus by Next-Generation Sequencing (NGS) (S1A Fig). We observed the best dynamic range at 2 µM RNP and chose this condition for the first stage of screening. In order to optimally inform rank order of gRNAs, we selected conditions that maximized the dynamic range of the assay and avoided any saturating signal, such that maximum editing at this stage was approximately 50%.

We proceeded to test the 243 *ADAR*-targeting gRNAs, utilizing a crRNA:tracrRNA format for the primary screen. We observed a large range in editing across gRNAs, ranging from ~0–50% editing (Fig 1C). Guide RNAs with very low editing included some with high bioinformatically-predicted efficiency scores, highlighting the importance of empirical evaluation in addition to *in silico* selection (S1B Fig). Of note, the top 10 most efficient gRNAs in this dataset are located within exon 2 (Fig 1D). Whether this observation reflects a bona fide increase in the susceptibility of this exon to be edited, particular sequence characteristics that affect editing rates, or statistical chance remains to be determined. The repair outcomes for a subset of gRNAs skewed toward in-frame mutations with a greater deviation between indel and frameshift mutation rates observed (S1C Fig, S2 Table). This highlights the need for empirical evaluation, ideally in cell types of interest, given that sequence context and additional biological factors will affect repair outcomes and thus the functional gene knockout rate.

Based on the results of the primary screen we selected 22 gRNAs with the highest rate of frameshift mutations, which are the most likely to disrupt protein function, for follow-up testing. In this initial validation and in subsequent characterization, gRNAs were tested in sgRNA format, since this is the most relevant format for a drug product. We evaluated the editing activity of the 22 selected gRNAs with a dose-titration of RNP that would provide a maximal dynamic range based on initial optimization experiments. In addition, to confirm that the relative editing efficiencies observed of specific gRNAs are not highly sensitive to different individuals we performed this follow up evaluation using primary human T cells derived from a different donor than in the initial primary screen. Editing efficiency of these candidate gRNAs were dose-dependent, consistent with the expected dynamic range (Fig 1E, S3 Table). Furthermore, the absolute value and rank-order of gRNA editing activity between gRNAs was similar between the primary screen and following evaluation (Fig 1F).

### Secondary screen of candidate gRNAs in primary human macrophages

**Fig 2:**
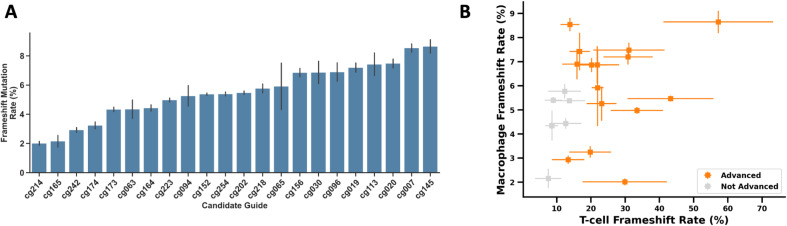
Guide RNA validation in human macrophages. A) Frameshift mutation rate for the 22 *ADAR*-targeting gRNAs chosen for follow-up in primary human macrophages. B) Comparison of frameshift mutation rates observed in primary human T cells vs. macrophages. Values for T cells are the same as shown in Fig 1F. Guide RNAs that were advanced for further validation are shown in orange. Data points represent the average of 3 biological replicates ±  S.D.

We further tested the 22 gRNAs in human macrophages as an orthogonal relevant ADAR-expressing cell type to identify gRNAs that are broadly active and perform similarly in distinct cell contexts. As in the primary screens with T cells, we observed a range of editing rates (Fig 2A, S4 Table). The maximum editing observed via this approach in primary macrophages was only ~9% but still enabled differentiation and rank ordering of gRNAs based on editing efficiency. Notably, we observed a strong positive correlation between editing rates in T cells and macrophages (Fig 2B). Two gRNAs were among the top performers in one cell type but showed lower editing in the other. The mechanistic explanation for this observation is unknown, but raises the possibility that particular gRNAs may have greater differences in editing rates between cell types (e.g., based on epigenetic factors or repair machinery differences). Based on the therapeutic approach it may be preferable to utilize either gRNAs with consistent rates between cell types or gRNAs with a propensity for cell type-specific outcomes.

### Characterization of ADAR protein loss associated with *ADAR* gene targeting

**Fig 3:**
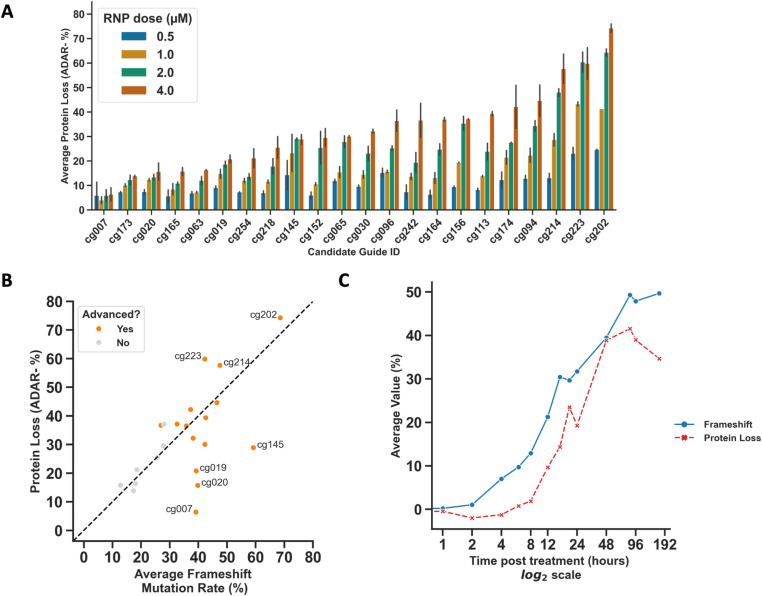
Loss of ADAR protein after editing with *ADAR*-targeting gRNAs. A) Loss of ADAR protein as measured by intracellular flow cytometry. Samples for flow cytometry were taken in parallel with gDNA collection in samples shown in Fig 1E. Data are plotted as mean ±  S.D. B) Comparison of the average frameshift mutation rate vs. protein loss observed in the secondary screen in human T cells. The average frameshift mutation rate and average protein loss are shown for the 4 µM RNP dose taken from Fig 1E (frameshift) and Fig 3B (protein loss) (Fig 1E) vs. average protein loss at the 4 µM RNP dose. C) Results of a kinetic experiment tracking the appearance of frameshift mutations and ADAR protein loss over time after T cells were treated with an *ADAR*-targeting RNP. For each time point after treatment, samples were taken for gDNA isolation and quantification of frameshift mutation by NGS, or for analysis of ADAR protein loss by intracellular flow cytometry. The average value for frameshift mutation and protein loss at each time point is plotted in blue and red, respectively. Data points represent the mean of 3 biological replicates.

We then investigated the effects of each gRNA on ADAR protein to confirm expected phenotypic effects based on genotypic outcomes. Using intracellular flow cytometry and NGS, we measured ADAR protein levels and editing rates, respectively, in matched samples. We observed a strong correlation between frameshift mutation rates and the fraction of cells that were negative for ADAR protein detection (Fig 3A–B, S5 Table, S6 Table). Surprisingly, the four gRNAs (cg007, cg019, cg020, cg145) targeting the most 5’ region of the coding sequence showed substantially lower ADAR protein loss compared to the frameshift mutation rate. Upon investigation of potential explanations, we noted that although there is no in-frame AUG start codon in a relevant window of the *ADA*R mRNA, there is an in-frame CUG codon downstream of these four gRNAs. The in-frame CUG could potentially serve as an alternative initiation codon with near-cognate efficiency [13–15]. We deprioritized these four guide RNAs (cg007, cg019, cg020, cg145) based on the attenuated protein loss and thus did not experimentally test this hypothesis. The observed discrepancy between genomic editing and protein loss does however highlight the value of empirical evaluation of editing activity in relevant cell types as well as inclusion of additional criteria beyond genomic position relative to the 5’ start site in gRNA identification (e.g., functional domains).

Next, to inform the likely timescale of biological consequences of *ADAR* gene knockout we assessed the kinetics of ADAR protein loss after gene targeting in primary human T cells using one of the candidate *ADAR* gRNAs. We observed protein loss ~6–12 hours after detection of frameshift mutations with relative protein loss and frameshift levels corresponding throughout the time course of the study (Fig 3C, S7 Table). These results are compatible with the time scale of protein turnover of exogenously expressed p110 ADAR protein that has been previously reported [16]. These data suggest that pharmacodynamic responses are likely to be induced rapidly after *ADAR* gene targeting, likely initiating within 24 hours or less.

### Characterization of candidate gRNAs

**Fig 4:**
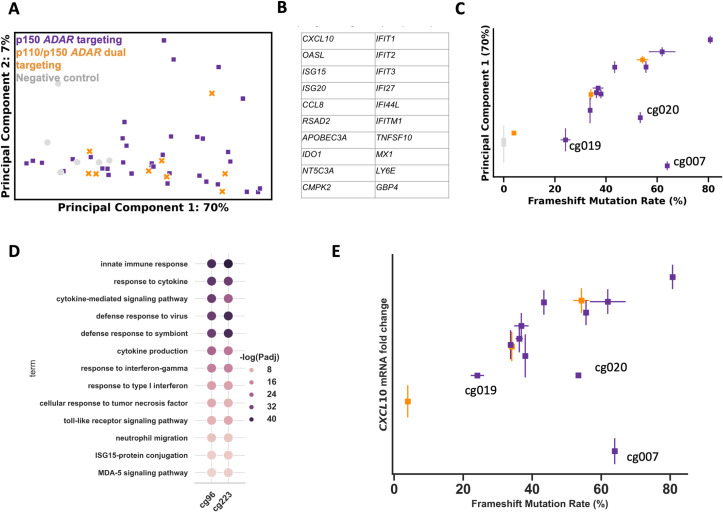
Immunological responses induced by *ADAR* targeting revealed by RNA-Seq analysis A) Comparison of p150 isoform specific targeting and p110/p150 dual isoform *ADAR*-targeting gRNAs as assessed by principal component analysis of RNASeq data. Points are shown in the first two principal components. B) The top 20 genes weighted in principal component 1 of the RNAseq principal component analysis. C) Principal component 1 values of RNAseq analysis displayed as a function of editing activity of gRNAs are displayed on a per sample basis. Guide RNAs located in the most 5’ region of ADAR are denoted (cg007, cg019, cg020). D) Upregulated gene ontology terms and statistical significance measured by -log_10_(adjusted-p-value) are displayed for representative gRNAs cg096 (p150 specific targeting) and cg223 (p110/p150 dual targeting). E) The fold-change of *CXCL10* mRNA measured by RNAseq is plotted as a function of frameshift editing rate.

A critical step for selection of a gRNA for a therapeutic candidate is characterization of the mechanism of action. We characterized the in vitro pharmacodynamic effects of the nominated candidate gRNAs identified via the primary and secondary screens using an RNAseq approach (see Methods). Based on the hypothesized therapeutic mechanism of action of ADAR knockout (i.e., induction of a type I interferon response), we utilized primary human macrophages as a highly relevant cell system. First we investigated whether different transcriptional responses are observed with the candidate gRNAs that target either p150 ADAR specifically versus gRNAs that target both p110 and p150 ADAR. We performed RNAseq on primary human macrophages treated with RNPs targeting either p150 ADAR, both isoforms of ADAR, or negative control genomic regions (i.e., safe harbor or intronic sequences). Significant transcriptional responses were associated with *ADAR*-targeting gRNAs compared to negative controls (See methods). In contrast, no substantial differences were observed in comparison of p150 and p110/150 *ADAR*-targeting gRNAs (Fig 4A, 4D, S8 Table). Inspection of the gene weighting of the first principal component revealed that the top 20 genes were primarily associated with an innate immune response (Fig 4B). Together these data suggest that in the context of non-developmental homeostatic conditions p150 loss mediates the bulk (>70%) of the response. To follow up on our prior finding that gRNAs 5’ of a putative non-AUG translational start site do not lead to ADAR protein loss, we included three such gRNAs (cg007, cg019, cg020) that had high indel frequencies in the primary screen in this analysis. Consistent with significantly attenuated ADAR protein loss associated with these gRNAs and the hypothesis that protein loss mediates the biological effects of *ADAR* targeting, these gRNAs did not induce a substantial transcriptional response compared to the negative control gRNAs (Fig 4C, S2B Fig). Next we assessed whether these transcriptional responses correlate with editing rate. For this analysis we evaluated only gRNAs targeting downstream of the putative alternative start site, excluding gRNAs cg007, cg019, cg020. Consistent with known ADAR biology, we observed a strong linear correlation ($r^{2}=0.914)$ between editing rate and transcriptional response (PC1) (Fig 4C).

To further investigate the nature of the transcriptional response induced by *ADAR* gene targeting we performed gene ontology analysis. Importantly, proximal RNA sensing pathways, including MDA5 signaling, are activated by ADAR targeting - indicative of effective target engagement. Additional terms consistent with the known biology of ADAR were highly enriched including general innate immunity and the specific hypothesized mechanism of action engaged by *ADAR* knockout (e.g., response to type I interferon, cytokine production). Supportive of this observation, mRNA levels of *CXCL10*, a canonical proximal type I interferon response gene, correlated strongly with *ADAR* editing rate (Fig 4E, S9 Table). Differential gene expression analyses of the gRNA with the highest observed editing efficiency further indicated that *ADAR* targeting leads to a dramatic transcriptional response consistent with gene ontology analyses (S2A Fig, S10–S11 Tables).

Off-target profiling is another important aspect of therapeutic gRNA development. We analyzed the 22 gRNAs described above with the commonly used MIT and CDF specificity scores (S12 Table) as well as alignment based off-target prediction tools Cas-OFFinder and CRISPOR (S1 and S13 Tables). None of the 22 gRNAs have a predicted off-target site with 1 or 0 mismatches within an exon. We assessed potential reactivity with ADAR paralogs, ADAR2 and ADAR3. None of the 22 gRNAs have predicted off-target sites with 4 mismatches or less that correspond to the coding sequences of ADAR paralogs (S14 Table). Of note, the gRNAs cg094, cg156, and cg233 were predicted to have particularly high specificity, having no predicted off-target sites with less than 3 mismatches.

Based on the collective findings from the primary screen, secondary screen, protein loss characterization, off-target analyses, and RNAseq-based analyses we report a shortlist of 12 candidate gRNAs suitable for therapeutic development (S1 Table). The final selection of which gRNA for a given therapeutic application should be informed by confirmation of activity using the therapeutic delivery method, confirmation of on-target activity in relevant cell types, off-target analyses in relevant cell types, and further mechanistic characterization in appropriate experimental models.

### Empirical evaluation of gRNAs targeting murine *Adar*

Finally, to support therapeutic translation we conducted similar gRNA screening in primary murine T cells to identify an appropriate murine surrogate. Although two of the human *ADAR* gRNAs tested in sgRNA format perfectly match the mouse genome, with one of these gRNAs in our final gRNA candidate list, it is likely that a therapeutic candidate gRNA would require a murine surrogate. The coding sequences for mouse *Adar* and human *ADAR* have 64.5% identity conservation which significantly constrains the number of species cross reactive gRNAs. We performed analogous gRNA evaluations in primary mouse T cells and observed a range of editing levels across gRNAs with a multiple high-efficiency murine gRNAs identified, similar to our findings in primary human cells. (S3A–3B Fig, S14 Table). These data provide a set of candidate gRNAs for use as a preclinical surrogate to support therapeutic development, the selection of which should be informed by the selection of the human therapeutic gRNA.

## Discussion

Here we identified and characterized high efficiency gRNAs targeting *ADAR,* providing a set of candidates that are suitable for therapeutic development. We performed a saturating screen for *ADAR*-targeting SpCas9 gRNAs with appropriate locations within the gene structure to induce loss of function and a minimally acceptable computationally predicted profile. Although this depth is unnecessary in many cases, in the context of therapeutic application it is important to maximize the likelihood of identifying a very high efficiency gRNA with a favorable off-target profile for subsequent optimization and development. These gRNAs may serve as a shortlist of candidate gRNAs for therapeutic development applications. The editing characterization performed will inform editing efficiency and effects using similar spCas9 constructs, but confirmatory data with the therapeutic modality and specific nuclease sequence will be necessary. We performed computational off-target analyses to inform the suitability of these gRNAs for potential therapeutic development. Empirical off-target analyses (e.g., site nomination and characterization assays) should be performed in the most biologically relevant cell type feasible for the target disease indication and with the exact nuclease construct being developed. As such, we did not perform empirical off-target analyses as part of the characterization here. Because we evaluated these gRNAs in multiple cell-based systems and in the context of electroporation and non-electroporation mediated delivery, it is likely that these gRNAs have utility across different SpCas9 delivery modalities. These gRNAs may have utility in multiple therapeutic areas in which *ADAR* knockout is the desired outcome. For example, there is strong biological rationale for *ADAR* targeting in the context of immuno-oncology. ADAR loss of function leads to an induction of a type I interferon response, which via other therapeutic approaches has been demonstrated to lead to a shift in the tumor environment towards an immune-permissive, anti-tumor state [17–20]. Based on the functional induction of a type I interferon response by ADAR KO and the known downstream cascading immunological responses that can result from type I interferon, it is reasonable to hypothesize that engagement of even a relatively small fraction of cells within the tumor microenvironment would be sufficient to remodel the tumor microenvironment and enhance anti-tumor immunity. Supportive of this notion, direct targeting of ADAR in tumor cells is sufficient to induce anti-tumor immunity, lead to tumor cell lethality, and can enhance the anti-tumor response to immune checkpoint blockade [4,21]. Furthermore, based on the biological function of ADAR to prevent constitutive recognition of self RNA, there is strong rationale that the effect of ADAR knockout in the tumor microenvironment would be observed in all cell types. It is likely that *ADAR* knockout in tumor infiltrating immune cells would have a similar, or perhaps even greater effect, based on the cells’ ability to efficiently produce cytokines. This is consistent with the pharmacodynamic responses we observed in *ADAR* targeted primary human macrophages. Further supportive of this hypothesis, ADAR loss in macrophages leads to an interferon response [22]. As such, it is tempting to speculate that these SpCas9 *ADAR*-targeting gRNAs could be effective in enhancing anti-tumor immune responses in the context of different therapeutic delivery modalities (e.g., lipid nanoparticles, viral vectors, RNP). More broadly, these *ADAR*-targeting gRNAs may even be useful as candidates for genome engineering of the endogenous *ADAR* locus (e.g., via HDR), applications of ADAR mediated RNA editing, or ADAR based programmable RNA sensors (e.g., RADARs) [23].

## Materials and methods

### In silico identification of gRNAs

A total of 553 candidate gRNAs were first identified by searching for all 20-mer protospacers with a canonical NGG protospacer adjacent motif (PAM) for SpCas9. We characterized these gRNAs by the location of the cut site in the p150 isoform of ADAR (CCDS1071), their inclusion in the p110 isoform (CCDS30879), and computationally predicted off-target activity, and homology to genomes of mouse, rhesus macaque, and cynomolgus monkey, species commonly used in preclinical evaluation.

Cut site index was calculated by taking the nucleotide to the left of the cut site for *Sp. Cas9*, or 4 base pairs away from the NGG protospacer adjacent motif and calculating its location in the coding sequence of CCDS1071. Codon index was determined via the cut site index. If the cut site was between two codons, the N terminal codon was used. Potential off-targets were determined using Cas-OFFinder with less than 3 mismatches, DNA bulge of 0, and RNA bulge of 0 and an NRG PAM [24]. Guide RNAs which had no off-target locations with less than 3 mismatches were classified as likely specific. Cas-OFFinder was also used to assess species homology. Guide RNAs which had perfect matches with NGG PAMs in their corresponding species were classified as homologous.

Protospacers were then filtered for cut sites before Alanine 980 and then either homology to the RheMac10 genome or gRNAs which were classified as likely specific and included within exon 2. This filtering resulted in 243 unique gRNAs (S1 Table). Three gRNAs have two targeting locations in the *ADAR* gene. To compare predicted activity with empirically measured activity, Doench scores were calculated for gRNAs selected for testing [25].

### Generation of RNPs

RNP components were thawed and maintained on ice during RNP assembly. Stocks of crRNA and tracrRNA, or sgRNA, were adjusted to HS-200 Buffer (10 mM Histidine, 100 mM Arginine, 200 mM NaCl, 5% sucrose (w/v), pH7.3). The gRNAs were refolded by incubating crRNA and tracrRNA at 70°C for 5 minutes then slowly cooled at room temperature for 10 minutes and then incubated on ice prior to complexation. RNP complexes targeting each gene were generated by mixing gRNA and Cas9 together on ice at a 1.2:1 molar ratio of gRNA:Cas. Cas9:gRNA mixtures were incubated at 30°C for 10 minutes, then moved immediately to ice.

### ADAR protein detection

ADAR protein levels were assessed after gene editing by intracellular flow cytometry. All flow cytometry readouts were done on the Attune NxT Flow Cytometer (ThermoFisher). Cells were first surfaced stained with an antibody panel discerning CD4+ and CD8+ T cells containing Aqua Live/Dead. After surface staining, cells were fixed and permeabilized using Cytofix/Cytoperm reagents (BD). Intracellular staining was then performed to detect ADAR protein levels, using an APC-conjugated anti-ADAR antibody (Abcam). Samples were then run on the flow cytometer and subsequent data output was analyzed using FlowJo^TM^ software. All antibody clones utilized are as follows: CD3 (UCHT1), CD56 (5.1H11), CD4 (OKT4), CD45 (HI30), CD19 (HIB19), CD8 (OKT-8), and ADAR (EPR7033).

### RNAseq

Libraries were prepared from total RNA isolated from primary human macrophages per manufacturer instructions via polyA selection for sequencing via 2x150bp, single indexing, and targeting 350M paired end reads with ≥80% of bases ≥Q30. Reads were pseudo-aligned to the human genome using kallisto with the pre-built human index, a default k-mer size of 31, and bootstrapping (with n = 100) (https://pachterlab.github.io/kallisto/about). The normalized transcript counts were further analyzed and tested for differential expression using DESeq2 [26]. All gRNAs were compared to an off-target control targeting an intronic region of the HPRT gene (5’ AATTATGGGGATTACTAGGA 3’). Two other negative controls were included: another off-target control targeting the AAVS1 gene (5’ GGGGCCACTAGGGACAGGAT 3’) and a gRNA which does not target the genome (5’ GCTGAAGCACTGCACGCCAT 3’). Transcript normalized counts were first aggregated at the gene level, then genes with an average of less than 10 counts per sample were filtered out of analysis. A blind variance stabilizing transformation was applied before principal component analysis with the top 500 weighted genes. Log fold changes were shrunk using the apeglm algorithm before differential expression testing was performed. Genes were classified as differentially expressed if the log2 fold change was less than 0.58 and the adjusted p-value was less than 0.05, only genes with a base mean of 3 are shown. Gene ontology analysis was performed with goseq [27] to analyze high level cellular response due to ADAR knockout via the genes classified as differentially expressed that increased in expression. P-values were Benjamini-Hochberg corrected for multiple hypothesis testing. Results from cg096 and cg223 a p150 specific targeting and p110/p150 dual targeting gRNA respectively, are shown (Table 10).

### Primary human T cell editing

RNP was complexed, snap frozen, and stored at -80C prior to use in T cell based assays. Primary human T cells were sourced from with Institutional Review Board approved human peripheral blood Leukopaks (STEMCELL Technologies) with data analyzed anonymously. Primary human T cells were thawed, then activated with 200 U/mL rhIL-2 (Peprotech) and 25 μL/mL ImmunoCult Human CD3/CD28 Activator (STEMCELL Technologies) and cultured for seven days before use in gRNA screening assays. On the day of gRNA screen execution, RNP was thawed and maintained on ice. T cells were counted and resuspended in nucleofection buffer P3 (Lonza) at a concentration of 25e6 cells/mL. T cells were gently mixed with RNP before transfer into a 96-well nucleofection cassette. Final number of T cells per nucleofection cassette well was 500,000 with an RNP concentration of 2 μM. Nucleofections were then executed using Lonza buffer P3 and pulse code EH115 in the 4D-Nucleofector Core Unit (Lonza). Following nucleofection, cells were briefly rested at room temperature before being plated in 200 uL of rhIL-2 supplemented T cell media in a 96-well round bottom plate (Falcon) and returned to culture. T cells were cultured for seven days after nucleofection, then gDNA was harvested from them using QuickExtract DNA Extraction Solution (Biosearch Technologies). Harvested gDNA was then used for NGS to determine editing efficiency of each *ADAR*-targeting RNP.

For the primary screen evaluation using 2-part guide RNA, annealing of crRNA:tracrRNA and formation of Cas9:RNA RNP complexes was done in singleton for each gRNA and nucleofected in triplicate wells using T cells from a single donor.

In experiments tracking the kinetics of gene editing protein loss, we used a previously peptide-assisted method similar to previously reported approaches [28–29]. RNPs were mixed with the peptide [H2N-krGMGAAGRKKRRQRRRPPAGTSVSLKKKRKVG{C(NPYS)}-amide], at a 4:1 RNP:peptide ratio by volume, then incubated at 25 °C for 2 hours in a thermocycler before immediately adding to cells. Stimulated T cells were washed and resuspended to 2.5 x 10^6^ cells/mL in human T cell media containing IL-2. 40 µL of cells were added to each well of a 96 well plate, followed by 10 µL of the RNP:peptide mixture to achieve 105 cells/well with 2 µM RNP and 8 µM peptide in a final volume of 50 µL. Cells were incubated with the RNPs for 1 hour before washing with media twice.. The cells were resuspended in a final volume of 200 µ L of human T cell media supplemented with IL-2 and incubated at 37 °C and 5% CO_2_. Cells were then harvested at the indicated time points for gDNA extraction or flow cytometry.

### Macrophage editing

Primary human CD14+ cells magnetically isolated from PBMC were thawed and differentiated into macrophages by culturing 7 days in media supplemented with 50 ng/mL M-CSF (Peprotech). Macrophages were plated in 96-well flat bottom, low attachment plates at 1 x 10^5^ cells per well. RNPs with either *ADAR*-targeting gRNAs or negative control gRNAs (targeting either AAVS1 as a safe harbor locus, an intronic region of HPRT, or BFP as a non-targeting control) were generated and used to treat the macrophage. The RNPs were delivered to M0 macrophages using a peptide-assisted method similar to the previously described approaches [28–29]. This approach was more tractable than nucleofection of macrophages and also allowed for evaluation of gRNAs delivered by a different method. Cells were co-incubated with 1 µM Cas9 complexed with *ADAR-*targeting gRNAs, plus 10 µM INF7-Tat-like peptide (GLFEKIEGFIENGWEGMIDGWYGYGRKKRRQRR, [30]) for 24 h. Media was removed and replaced with fresh supplemented media and cells were returned to culture for another 48h. gDNA was extracted from cells using the QuickExtract DNA Extraction Solution (Biosearch Technologies) for NGS readout.

### Mouse T cell editing

Whole spleens were harvested from six week old BALB/c mice (Charles River). Mice were euthanized by CO_2_ inhalation with another secondary method of euthanasia performed subsequently (e.g., cervical dislocation, removal of a major organ, or monitored exposure to 20 minutes of room air). Animal protocols were approved by the Protagonist Therapeutics Institutional Animal Care and Use Committee (IACUC). Spleens were manually dissociated and passed through a 70 μM strainer (Fisherbrand). Red blood cell lysis was performed on the cell isolate by resuspending cells in RBC lysis buffer (Sigma-Aldrich) and incubating for 3-5 minutes at room temperature. T cells magnetically isolated (Stemcell Technologies Easy Sep mouse T cell isolation kit (cat# 19851A) per manufacturer’s instruction and resuspended in culture media (RPMI-1640 (Gibco) containing 10% heat inactivated FBS (HyClone), 1% PenStrep (Gibco), 55 mM 2-mercaptoethanol (Gibco), 200 mM GlutaMAX (Gibco), 100 mM Sodium Pyruvate (Gibco), 10 mM HEPES (Gibco), 1x MEM NEAA (Gibco)) and activated with 60 U/mL of recombinant mouse IL-2 (R&D Systems) and Dynabeads Mouse T-activator CD3/CD28 (Gibco). Mouse T cells were cultured at 37°C in a humidified atmosphere with 5% CO2 for seven days prior to nucleofection. Nucleofections were then executed using Lonza Buffer P3 and pulse code DN100 in the 4D-Nucleofector Core Unit (Lonza).

### Next-gen sequencing

Next-generation sequencing library preparation followed standard amplicon sequencing workflows. Primers for PCR1 were designed using Primer 3 for amplification of 300-450 bp regions, with Illumina-compatible adapters added [31]. PCR1 used Q5 Hot start High-Fidelity 2X Master Mix (NEB, M0494L), 500 nM of each primer, and ~100 ng of genomic DNA in a total reaction volume of 20 µL(4). PCR1 thermocycling conditions were: 1 cycle for 1 min at 98°C, 35 cycles for 10 s at 98°C, 20 s at 60°C, 30 s at 72°C, and 1 cycle for 2 min at 72°C, followed by holding at 4°C. PCR1 products were diluted 100x with nuclease-free water and used as template for a second PCR. PCR2 was performed with xGen UDI indexing and adapter primers (IDT). PCR2 used Q5 Hot start High-Fidelity 2X Master Mix (NEB, M0494L), 500 nM of each primer, and 8 µL of diluted PCR1 as template in a total reaction volume of 20 µL. PCR2 thermocycling conditions were: 1 cycle for 1 min at 98°C, 12 cycles for 10 s at 98°C, 20 s at 60°C, 30 s at 72°C, and 1 cycle for 2 min at 72°C, followed by holding at 4°C. PCR2 products were pooled and cleaned up with AMPURE XP beads (Beckman Coulter) using a 0.7:1 volume ratio (bead:PCR product). Proper PCR product size was assessed with a Fragment Analyzer (Advanced Analytical) or by agarose gel electrophoresis, and libraries were quantified by qPCR (QuantStudio 6 Flex, Applied Biosystems). Libraries were sequenced on either an Illumina MiSeq or NextSeq sequencer, with 20% PhiX spike-in.

### NGS data analysis

FastQ files were checked for quality using FastQC (https://www.bioinformatics.babraham.ac.uk/projects/fastqc/) and MultiQC (https://multiqc.info/citation/) [32]. Trimming, alignment and quantification of editing was performed with CRISPResso2 v2.2.10 [33]. The trimmomatic command ILLUMINACLIP:trimmomatic_fastas/TruSeq3-PE-2.fa:2:30:10:2:true MINLEN:75 was used. For base editing samples a quantification center of -15 with a quantification window of 10 was used, whereas a quantification center of -3 and a window of 1 was used for non-homologous end joining. For all samples the minimum homology accepted was 70. For base editor samples the following parameters were added--base_editor_output--conversion_nuc_from A--conversion_nuc_to G. Summary data was compiled from CRISPResso output files and tabulated for downstream analysis.

### Expanded off-target prediction for guide RNAs advanced to secondary screen

Expanded off-target prediction was completed for all 22 gRNAs which advanced beyond the initial primary screen. Both the commonly used in silico off-target predictors, CFD and MIT specificity scores were compiled [25,34]. Additionally, comprehensive off-target information for all off-targets with less than 4 mismatches in intergenic or exonic sequences (excluding intronic off-targets, as classified by CRISPOR) was included from CRISPOR v5.2 using the default hg19 genome [35]. This includes information on the locus of the off-target and each off-target’s respective MIT and CFD scores

## Supporting information

S1 Fig A) Editing rates of two ADAR-targeting gRNAs (cg065 and cg223) and a negative control non-targeting gRNA are displayed as a function of RNP dose (represented as concentration). B) Average indel editing rate observed in the primary screen in human T cells displayed as a function of the computational on-target Doench efficiency score. C) Average frameshift editing rate per gRNA is displayed as a function of average indel editing rate. Guide RNAs with (yellow) or without (blue) a significant deviation observed between frameshift and indel rates are highlighted.
(TIFF)

S2 Fig A) RNAseq differential gene expression analysis of the ADAR-targeting gRNA cg096 versus negative control gRNA is displayed as a volcano plot. The highest editing efficiency within the evaluated set was observed with cg096 and thus is displayed here as an example. B) Principal component 1 values are displayed as a function of genomic position with the *ADAR* locus. The position of the location of an in-frame CUG codon is marked with a dotted red line. Guide RNAs that target either only the p150 ADAR isoform (blue) or both the p110 and p150 isoforms of ADAR (orange) are highlighted.
(TIFF)

S3 Fig A) The average frameshift editing rate observed in primary mouse T cells with murine *Adar*-targeting gRNAs is displayed. Average values reflect values of 3 technical replicates. B) Average indel editing rate observed in primary mouse T cells displayed as a function of the computational on-target Doench efficiency score is displayed.
(TIFF)

S1 Table Human *ADAR*-targeting guide RNA annotations. (XLSX)

S2 Table Human *ADAR*-targeting guide RNA editing data from primary screen using 2-part guide RNA. (XLSX)

S3 Table Human *ADAR*-targeting guide RNA dose response editing data. (XLSX)

S4 Table Human *ADAR*-targeting guide RNA editing data in primary human macrophages. (XLSX)

S5 Table Human *ADAR*-targeting guide RNA editing and protein loss data. (XLSX)

S6 Table Summary data of candidate human *ADAR*-targeting guide RNAs. (XLSX)

S7 Table Human *ADAR*-targeting guide RNA editing and protein loss kinetics data. (XLSX)

S8 Table Human *ADAR*-targeting guide RNA editing and principal component analysis data. (XLSX)

S9 Table *CXCL10* expression data associated with candidate human *ADAR*-targeting guide RNAs. (XLSX)

S10 Table RNAseq differential gene expression data. (XLSX)

S11 Table RNAseq gene ontology analyses. (XLSX)

S12 Table Off-target analysis scores of candidate human *ADAR*-targeting guide RNAs. (XLSX)

S13 Table Candidate human *ADAR*-targeting guide RNA off-target analysis data. (XLSX)

S14 Table Murine *Adar*-targeting guide RNA annotations and editing data. (XLSX)
