# Supplementary figures and images for: Systematic identification and characterization of high efficiency Cas9 guide RNAs for therapeutic targeting of ADAR
Source: PLoS One. 2025 Feb 24;20(2):e0317745. doi: 10.1371/journal.pone.0317745 (PMC11849909; doi:10.1371/journal.pone.0317745)

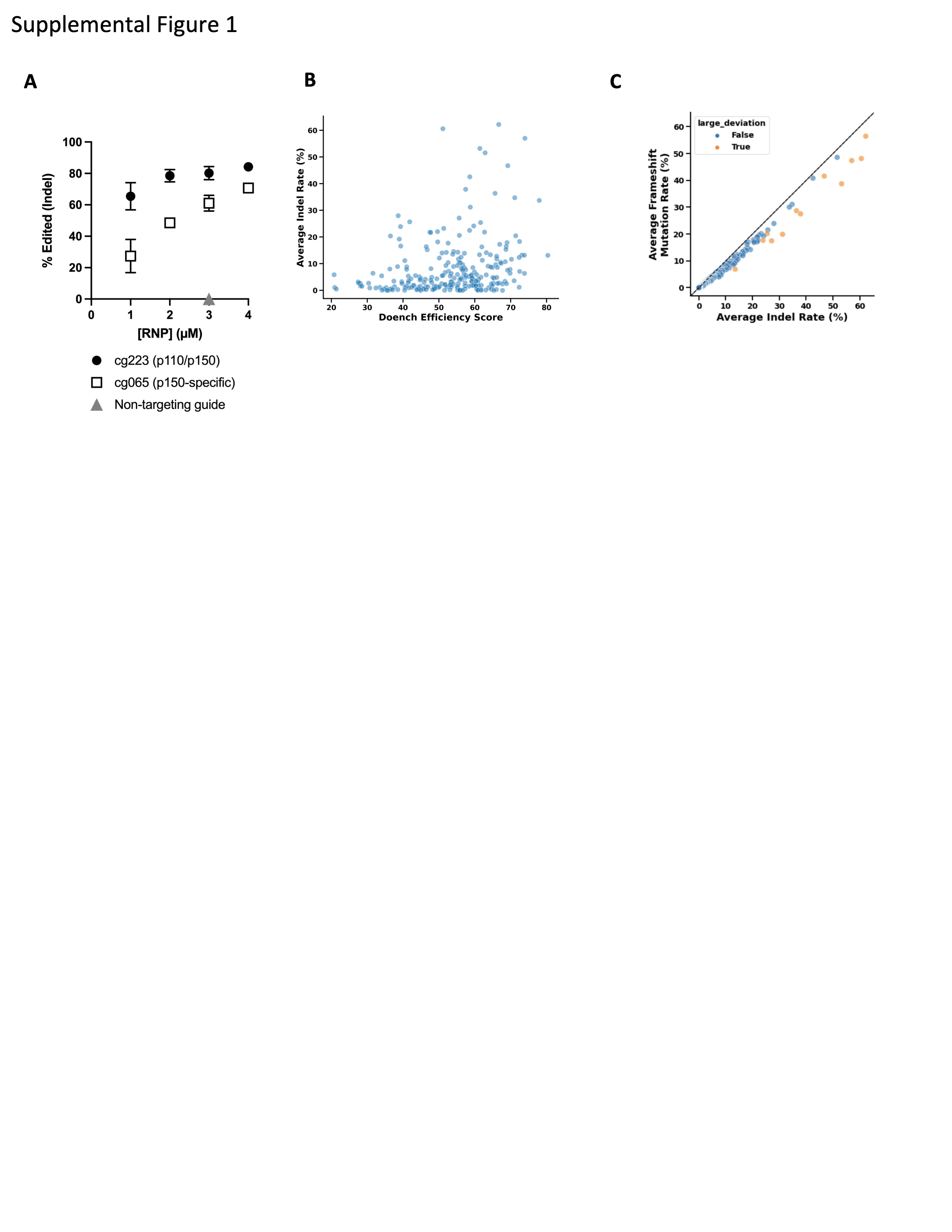

Supplement: S1 Fig — A) Editing rates of two ADAR-targeting gRNAs (cg065 and cg223) and a negative control non-targeting gRNA are displayed as a function of RNP dose (represented as concentration). B) Average indel editing rate observed in the primary screen in human T cells displayed as a function of the computational on-target Doench efficiency score. C) Average frameshift editing rate per gRNA is displayed as a function of average indel editing rate. Guide RNAs with (yellow) or without (blue) a significant deviation observed between frameshift and indel rates are highlighted. (TIFF) [file pone.0317745.s001.tiff]

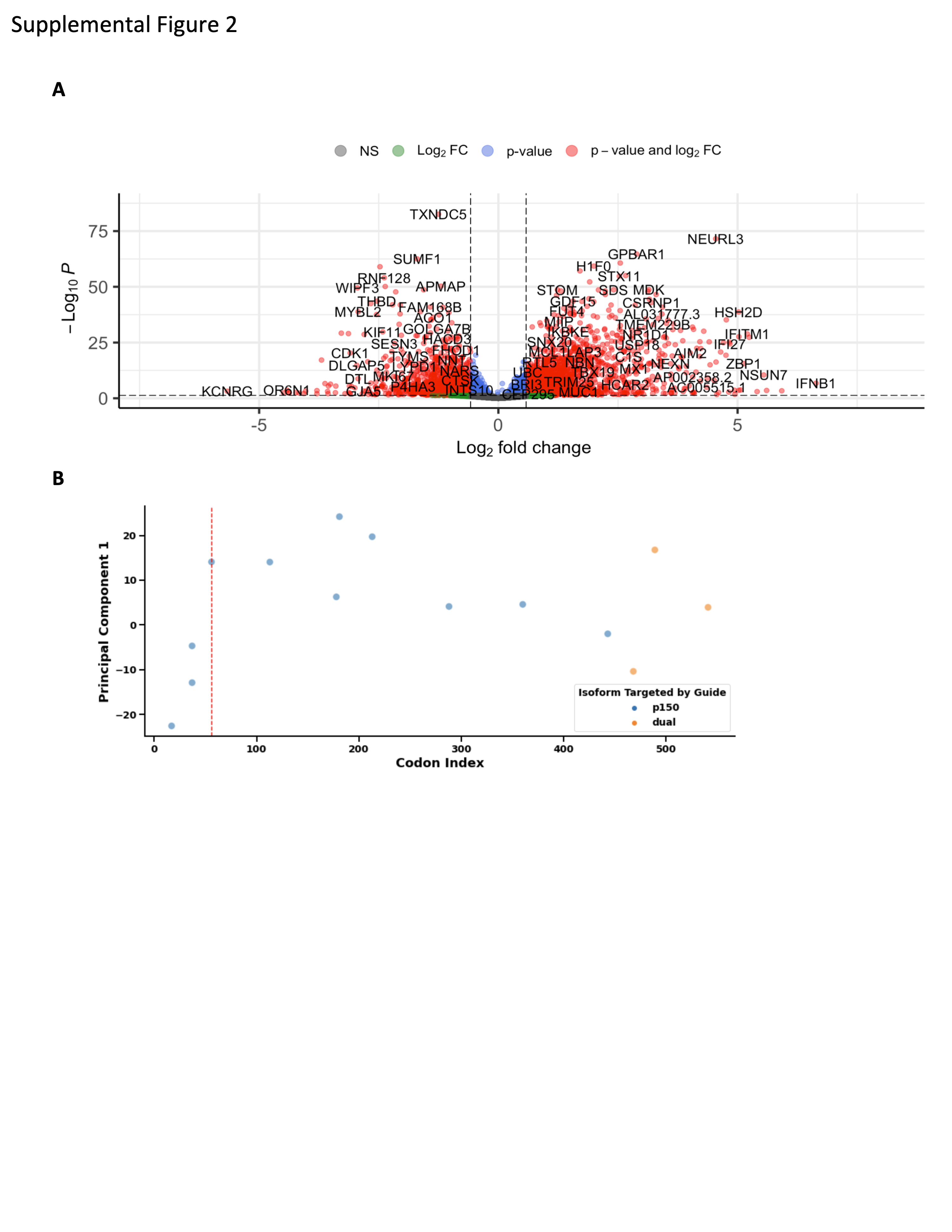

Supplement: S2 Fig — A) RNAseq differential gene expression analysis of the ADAR-targeting gRNA cg096 versus negative control gRNA is displayed as a volcano plot. The highest editing efficiency within the evaluated set was observed with cg096 and thus is displayed here as an example. B) Principal component 1 values are displayed as a function of genomic position with the ADAR locus. The position of the location of an in-frame CUG codon is marked with a dotted red line. Guide RNAs that target either only the p150 ADAR isoform (blue) or both the p110 and p150 isoforms of ADAR (orange) are highlighted. (TIFF) [file pone.0317745.s002.tiff]

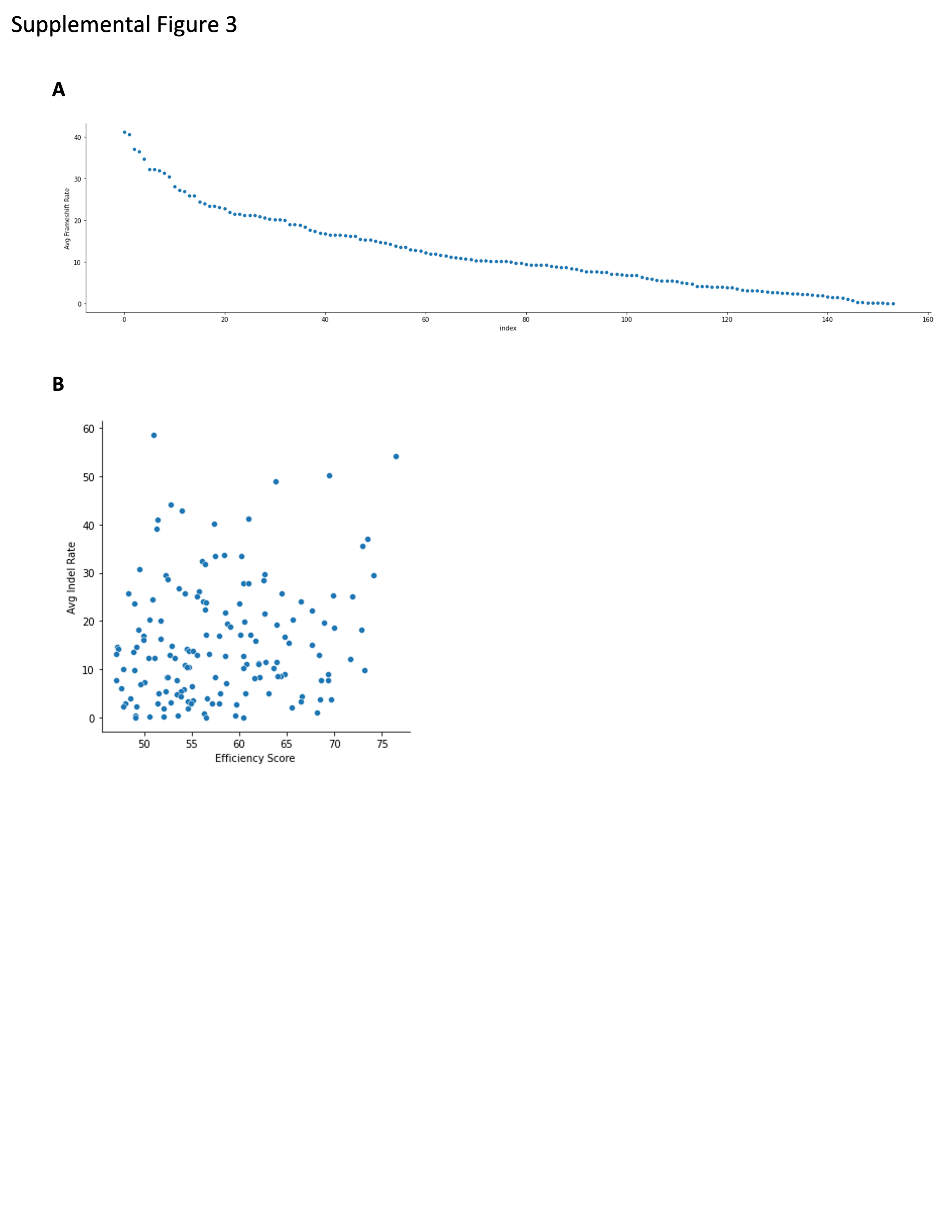

Supplement: S3 Fig — A) The average frameshift editing rate observed in primary mouse T cells with murine Adar-targeting gRNAs is displayed. Average values reflect values of 3 technical replicates. B) Average indel editing rate observed in primary mouse T cells displayed as a function of the computational on-target Doench efficiency score is displayed. (TIFF) [file pone.0317745.s003.tiff]
